# Effectiveness of "Centaurea behen" root on quality of life in patients with systolic heart failure: A randomized clinical trial

**DOI:** 10.34172/jcvtr.2023.31619

**Published:** 2023-03-16

**Authors:** Saeid Yousefi, Hassan Ahangar, Mohsen Bahrami, Mohammad Kamalinejad, Alireza Yaghoubi, Hosein Azizi

**Affiliations:** ^1^Department of Iranian Traditional Medicine, School of Medicine, Zanjan University of Medical Sciences, Zanjan, Iran; ^2^Department of Cardiology, Mousavi Hospital, Zanjan University of Medical Sciences, Zanjan, Iran; ^3^Department of Pharmacognosy, Faculty of Pharmacy, Shahid Beheshti University of Medical Sciences, Tehran, Iran; ^4^Heart Valve Disease Research Center, Rajaie Cardiovascular Medical and Research Center, Iran University of Medical Sciences, Tehran, Iran; ^5^Research Center of Psychiatry and Behavirol Sciences, Tabriz University of Medical Sciences, Tabriz, Iran

**Keywords:** Centaurea Behen, Heart Failure, Quality Of Life, Randomized Clinical Trials, Traditional Medicine

## Abstract

***Introduction:*** The effect of Centaurea behen (Cb) on patients with systolic heart failure is not known academically. This study was conducted to evaluate the effect of Cb on improving the quality of life (QoL) and echocardiographic and biochemical blood parameters in patients with systolic heart failure.

***Methods:*** This study was a parallel double-blind, placebo-controlled randomized trial of 60 patients with systolic heart failure, was conducted from May 2018 up to August 2019. Intervention group received 150 mg twice daily Cb capsules for two months + Guideline directed medical therapy (GDMT), and control group received GDMT + placebo capsules for two months. The main aim of the present study were to assess the QoL based on the 6-minute walk test (6MWT) and the Minnesota living with heart failure questionnaire (MLHFQ). Independent T-test, paired T-test, and ANOVA were used for the analysis.

***Results:*** At the beginning of the present study there were no significant differences between study groups in terms of QoL and clinical results. After treatment, the average values of QoL based on MLHFQ and 6MWT instruments were significantly improved 15.5 and 36.18, respectively (*P*<0.05).

***Conclusion:*** Based on the MLHFQ, and 6MWT tests, the consumption of Centaurea behen root extract was associated with significant improvement in the quality of life of patients with systolic heart failure.

## Introduction

 Heart failure (HF) is a condition in which the heart fails to fill the ventricles with blood or pump blood out of the ventricles and its main manifestations are fatigue and shortness of breath.^1^ It has been proven that ischemic heart disease is the most important risk factor of HF.^2^ Its prevalence is about 2% in people over 65 years of age, which, by the way increases with age and is associated with an absolute mortality of 50% in 5 years from the date of diagnosis.^1^ HF clearly causes reduction in health related quality of life (HRQoL), especially in terms of vital and physical function, and drug therapy cannot effectively alleviate it.^1^ The Quality of Life (QoL) is severely impaired in chronic heart failure (CHF) compared to other chronic diseases. Furthermore, despite the reduction in the duration of CHF treatment, the cost of treatment amounts to 1-2% of the total health budget.^3^ Heart failure is a final usual pathway of cardiovascular disease and despite the important therapeutic advances, morbidity and death are high and puts major burden of the health care.^4^ According to the American College of Cardiology Foundation/ American Heart Association (ACCF/ AHA) guidelines, HF is based on ejection fraction (EF) and is divided into two groups: HF with preserved EF (HFpEF) and HF with reduced EF (HFrEF). HFrEF is determined on the basis of clinical diagnosis and EF ≤ 40%.^1^

 The Centaurea behen Linn. (Cb), [Family Asteraceae/Compositae], which is commonly known as Bahman Sefid, is native to Iran.^5,6^ It has been used as an food plant,^7^ and also, as a medicine in the Iranian Traditional Medicine as a cardiotonic, aphrodisiac, sedative, anti-flatulent, and also has been used in treatment of jaundice, urinary stones, body inflammations, menstrual irregularities,^8^ cystic fibrosis,^5^ nervous system disorders,^9^ stomach problems,^10^ as well as weight increasing agent,^11^ analgesic and mood elevator since ancient times.^12^ The active ingredients of the plant include flavonoids,^8,13^ phenols, saponins, tannins, phytosterols, carbohydrates,^8^ taraxaestrol acetate, taraxaestrol myristate, inulin, alkaloids (bahamine), germacrine D, hexadecanoic acid, spathulenol, phytol,^14^ aguerin B, cynaropicrin, grosshemin, circimaritin, desacyl synaropicrin, naringenin, solstitialine A, 4β,15-dihydro-3-dehydrosolstitialin A diacetate and 4β, 15-dihydro-3-dehydrosolstitialin A monoacetate,^15^ ketone,^16^ luteoline,^17^ and some others.

 Previous research work on Cb products have shown various properties that may be effective in preventing the progression of HF, including properties such as antioxidant,^5,18^ hepatoprotective,^18^ antianxiety,^8^ cytotoxic, antiangiogenic, anti-cancer, antimicrobial,^9^ apoptotic,^13^ and anti-fungal.^19,20^

 Previous Cb trials have also shown in vivo (in mice) or in vitro effective results on the liver,^18^ central nervous system,^8^ human umbilical vein endothelial cells (HUVECs) and human breast/ovarian cancer cell lines.^9^ Therefore, this double-blind, placebo-controlled clinical trial was conducted with the aim of investigating the effects of standard Cb root extract as the first study of Cb on increasing QoL and improving heart function in patients with HFrEF. Minnesota Living with Heart Failure Questionnaire (MLHFQ), 6-minute walk test (6MWT), several echocardiographic parameters and control laboratory tests were used.

## Materials and Methods

###  Study design and setting

 This study was the phase III, randomized, double-blind, placebo-controlled trial to evaluate the effects of Cb root extract capsule (150 mg twice daily) versus placebo in a 60-day study period on the QoL of patients with systolic heart failure.

**Figure 1:**
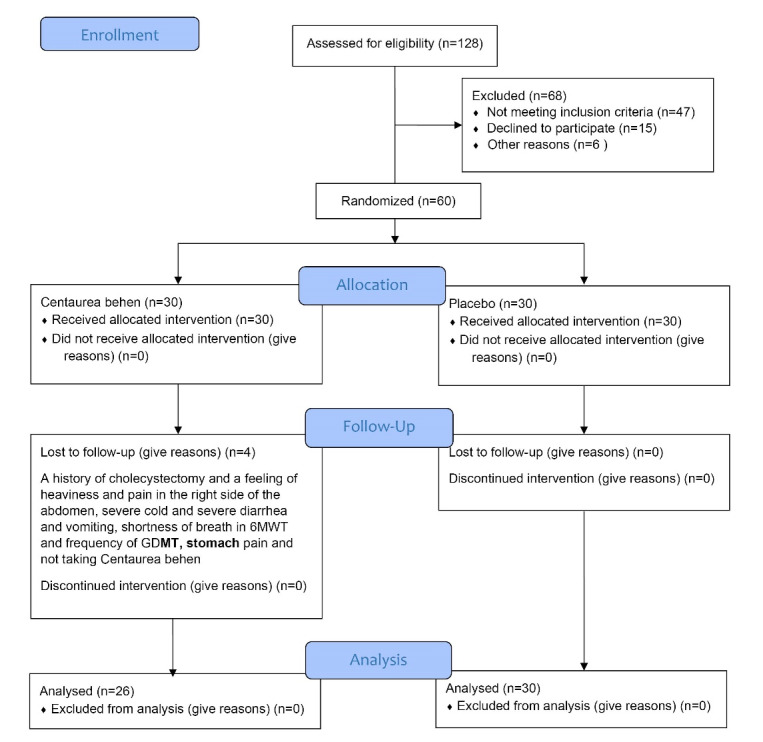
CONSORT 2010 Flow Diagram of the research. Abbreviations: 6MWT, 6-minute walk test; GDMT, Guideline directed medical therapy

 The study population was patients with systolic heart failure, in the Cardiovascular Clinic of Zanjan University of Medical Sciences in Zanjan, Iran. Participant’s recruitment was performed from May 2018 to August 2019. During this period, 128 patients with systolic heart failure were assessed for eligibility, and 68 cases were excluded (47 patients were not eligible for the study, 15 patients refused to participate, and 6 patients were not included for other reasons). The age distribution for the participants was 40 to 75 years old and 53% were male. Overall, a total of 60 patients with systolic heart failure (30 patients in each group) who had medical records, were randomly selected into two groups of intervention and control (Figure 1).^21^ All patients in the intervention and control groups continued to receive Guideline directed medical therapy (GDMT), for systolic heart failure during a 60-day study period. The most common drugs used as GDMT for systolic heart failure in participants were Carvedilol, Hydralazine, Atorvastatin, Clopidogrel, Amlodipine, Rosuvastatin, Nitroglycerin, Losartan, Metoprolol, Spironolactone, Warfarin, Digoxin, Acetylsalicylic Acid, Furosemide, Valsartan, Hydrochlorothiazide, Diltiazem, Eplerenone, Simvastatin, Nicorandil, Lisinopril, Amiodarone, Fenofibrate_. _

 Based on the pilot study, the sample size was determined with the mean ± standard deviation for the intervention and control groups, 20 ± 12 and 30 ± 14, respectively, considering the QoL as the outcome variable. Considering α = 0.05, β = 0.2 and 5% compensation for the loss to follow-up cases, 30 cases in each group (a total of 60 patients) were selected.

###  Eligibility criteria

 The inclusion criteria were: 1) Systolic heart failure with the ejection fraction (EF) ≤ 40% 2) Ages between 40 to 75 years old 3) Receiving permanent and continuous treatment for heart failure within last 6 months and continuing to take “heart medications” prescribed by their cardiologist during the study; and 4) Patients with the functional classification of New York Heart Association (NYHA) I and II.

 The exclusion criteria were: 1) Pregnancy or breast feeding 2) History of malignancy 3) Considerable renal insufficiency 4) History of hypersensitivity reaction to Cb root 5) Chronic inflammatory disease 6) History of collagen vascular disease 7) Severe liver disease 8) Acute infectious disease 9) Developing of decompensated heart failure during the research; and 10) The patient’s disinclination to continue being involved in the research or being unable or eager to follow-up.

###  Randomization, blinding and follow up

 Eligible patients were randomly divided into two parallel groups of intervention and control (30 participants in each group). Randomization was done using a table of random numbers. Generation of the random allocation sequence, intervention and placebo coding, and participants’ assignments were the responsibility of a statistical consultant who was not blinded to the study.

 Each patient was assigned a unique code during the study period. Cb and placebo capsules had the similar shape, color and packaging but with different codes. All patients, healthcare providers, and investigators were blinded to the intervention-control groups. Patients in the intervention group received 150 mg of Cb root extract (active ingredient) capsules twice daily for 60 days. The study follow-up period was 60 days and participants were visited twice a month during this period at the clinic to ensure the appropriate usage of the drug and high compliance, record vital signs and appraise the study progress. At each follow-up, patients were evaluated by taking blood pressure in both arms, measuring heart rate, and asking open-ended questions about the incidence of drug side effects or allergic reactions. There were no complaints about the above mentioned items. In order to measure the probability of liver damage in the participants, serum enzymes AST, ALT and ALP were considered. Elevated levels of these sensitive markers indicate cellular leakage due to loss of functional integrity of the cell membrane in hepatocellular injury.^18^ The results of these tests can suggest the absence of liver cell damage in this study.

###  Drug preparation 

 First, to prepare Cb root extract capsules, the plant was thoroughly washed to remove contaminants. Then 100 grams of dried Cb root was poured into a beaker with 1.5 liters of water and boiled on a flame for 15 minutes. The beaker was cooled to room temperature and the solution was filtered. In order to extract the Cb concentrate, a rotatory evaporator was used to purify and concentrate the solution. Due to the fact that 10% of the weight of primary Cb root was dry extract, corn starch was added as an excipient. Finally, 500 mg capsules of Cb concentrate were prepared. Thus, each 500 mg Cb capsule contained 150 mg of active ingredient plus 350 mg of excipient, as stated.

 To prepare equal medication with neutral effect in control group, 500 mg capsules with corn starch was provided. In both the intervention and control groups, the capsules were similar in terms of shape and color, and due to the double-blind rule of the study, they were identified with the corresponding code. Finally, the capsules were prepared in plastic capsule bottles containing 60 capsules, which were similar in shape, color and label in both groups.

 All steps of drug preparation were carried out in the School of Pharmacy of Shahid Beheshti University of Medical Sciences (SBMU) with the institutional herbarium approval number SBMU-8097.

###  Total Phenol Content (TPC) 

 To measure the TPC, 1 mL of Gallic acid in separate concentrations of 20, 40, 60, 80, and 100 µg/mL were mixed for ten minutes with 5 mL of Folin-Ciocalteu reagent (1:10 dilution in distilled water). Then 4 mL of aqueous Na_2_CO_3_ (7.5 mg/mL) was added.

 Then, after incubating the resulting mixture for 30 minutes in a dark place at room temperature, the rate of absorption at a wavelength of 765 nm was obtained using a spectrophotometer.

 After obtaining the Gallic acid calibration curve with the above concentrations, the calculation was repeated with 1 mL of the extracted solution (30 mg/mL) as a substitute for Gallic acid. Finally, TPC was obtained using Gallic acid standard curve.

###  Total Flavonoid Content (TFC) 

 To measure the TFC, 2.5 mL of rutin solution in separate concentrations of 20, 40, 60, 80, and 100 µg/mL, were mixed for forty minutes with 2.5 mL of aluminum chloride in 80% ethanol solution (20 mg/mL). Then the absorption rate at a wavelength of 415 nm was achieved using a spectrophotometer. Finally, after obtaining the rutin calibration curve with the above concentrations, the calculation was repeated with 2.5 mL of the extracted solution (30 mg/mL) as a substitute for rutin, and the TFC was obtained using the rutin standard curve.

 The total flavonoid and total phenolic contents were 0.21 mg/capsule and 0.91 mg/capsule, respectively.

###  Outcome

 The primary outcome of the study was an improvement in QoL at the end of the 60 days of intervention. Qol was measured using the MLHFQ parameters and 6MWT results. The secondary outcomes were also the changes in echocardiographic parameters and blood biochemical levels at the end of the 60 days of intervention.

 At the beginning of the study, a cardiologist measured the heart function of all participants through conventional standard two-dimensional and Doppler transthoracic echocardiography (Vivid 7; GE), and at the end of 60 days of the intervention, the same cardiologist performed echocardiography of all participants.

 Left ventricular (LV) function was evaluated with the following parameters:

1. Left ventricular ejection fraction (LVEF) was calculated by Simpson’s echocardiography (EFS) method.
2. Ejection time (ET), isovolumic relaxation time (IVRT) and isovolumic contraction time (IVCT).
3. Left Ventricular Myocardial Performance Index (LV MPI) using (IVRT + IVCT)/ET formula.
4. The ratio between the early diastolic Trans mitral flow velocity (E) and the mitral early diastolic annular velocity (e’), (mean E/e’).

Right ventricular (RV) function was measured by fractional area change (FAC) using the formula (RV area in diastole-RV area in systole)/ (RV area in diastole) × 100.

###  Statistical analysis

 The IBM SPSS Statistics software (Version 25) was carried out for data analysis. Chi-square test was used to analysis categorical variables. *Independent T-test* was used to compare quantitative variables between-group and *paired T-test* was used for within group comparisons. The *one-way ANOVA *was applied to determine Between-Subjects Effects. Data normality was checked by Kolmogorov-Smirnov test. In all tests the *P* value < 0.05 was considered significant level.

## Results

 Total number of 56 patients participated in the study. Demographic and medical history of the participants are presented in Table 1. The two groups had similar initial characteristics. There was no statistically significant association regarding demographic characteristics, pre-existing disease history, comorbidities, body mass index (BMI), and New York Heart Association (NYHA) Functional Classification between groups before intervention (*P* < 0.05).

**Table 1:** Demographic and baseline characteristics of the participants

**Variables**		**Treatment** **n (%)**	**Placebo** **n (%)**	**Total**	***P*** ** value**
Gender					0.493
Male		12 (46.2)	18 (60)	30 (53.5)	
Female		14 (53.8)	12 (40)	26 (46.5)	
Age (years)					0.328
40-60		10 (38.5)	14 (46.7)	24 (42.8)	
61-75		16 (61.5)	16 (53.3)	32 (57.2)	
Marital status					0.037
Married		18 (69.2)	26 (86.7)	44 (78.6)	
Widow/er		8 (30.8)	4 (13.3)	12 (21.4)	
Any disease history					0.669
Yes		11 (42.3)	18 (60)	29 (51.8)	
No		15 (57.7)	12 (40)	27 (48.2)	
Smoking					0.413
Current smoker		1 (3.8)	3 (10)	4 (7.2)	
Quit smoking		8 (30.8)	7 (23.3)	15 (26.7)	
No		17 (65.4)	20 (66.7)	37 (66.1)	
Comorbidity					
Hypertension		4 (15.4)	7 (23.3)	11 (19.6)	0.152
Diabetes		10 (38.5)	6 (20)	16 (28.6)	0.186
BMI ( ≥ 30) (kg/m^2^)		8 (30.8)	9 (30)	17 (30.4)	0.956
Hypothyroidism		1 (3.8)	0 (0)	1 (1.8)	0.122
NYHA class	I	6 (23.1)	9 (30)	15 (26.8)	0.671
	II	20 (76.9)	21 (70)	41 (73.2)	
Regular Exercise	Yes	12 (46.2)	9 (30)	21 (37.5)	0.175
	No	14 (53.8)	21 (70)	35 (62.5)	

Abbreviations: BMI, body mass index; NYHA, New York Heart Association. *P* < 0.05 statistically significant.

Table 2 indicates quality of life using the 6MWT and MLHFQ in the study groups before and after intervention. As a result, Cb decreased the MLHFQ by an average of 15.5 points (the mean average of MLHFQ was 37.5 at the beginning of the study and 22 at the end of the study). There were statistically significant differences in MLHFQ score between and within the groups after intervention (*P* < 0.05).

**Table 2:** Comparison of 6MWT and MLHFQ in the study groups

**Variables**	**Treatment**	**Before**		**After**		***P*** ** value (paired)**
		**Mean**	**S.D**	**Mean**	**S.D**	
MLHFQ	Centaurea behen	37.5	15.42	22.00	12.69	< 0.001
	placebo	40.16	17.5	25.93	16.63	< 0.001
*P*-value (between)		0.724		0.051		
6MWT,m	Centaurea behen	349.08	135.79	385.26	156.51	0.046
	placebo	351.1	114.78	376.66	115.24	0.091
*P*-value (between)		0.012		0.001		

Abbreviations: MLHFQ, Minnesota living with heart failure questionnaire; 6MWT, the 6-minute walk test. *P* < 0.05 statistically significant.

 Furthermore, Cb increased the 6MWT value by an average of 36.18 points (average value of 349.08 at the beginning of the study and 385.26 after the intervention). Likewise, there were statistically significant differences of the 6MWT score between and within groups after intervention (*P* < 0.05). However, there was no significant difference within placebo group before and after the treatment (*P* = 0.091).

Table 3 shows mean and standard deviation of blood biochemical levels and echocardiographic parameters by treatment type. It was found that there were no statistically significant differences in blood biochemical levels and echocardiographic parameters. Although a slight difference was observed in right ventricular functional area change (RV FAC) and left ventricular myocardial performance index (LV MPI) between groups which was not statistically significant.

**Table 3:** Blood biochemical levels and echocardiographic variables of participants at the beginning and end of the study

**Variables**	**Treatment**	**Beginning**		**End**		***P*** ** value (within)**
		**Mean**	**S.D**	**Mean**	**S.D**	
AST, U/L	Centaurea behen	23.15	7.75	27.12	9.32	0.001
	placebo	25.37	10.11	26.37	8.86	0.830
*P*-value (between)		0.375		0.861		
ALT, U/L	Centaurea behen	23.46	13.35	22.73	8.66	0.000
	placebo	28.73	12.47	25.10	10.51	0.212
*P*-value (between)		0.147		0.291		
ALP, U/L	Centaurea behen	223.27	95.32	210.92	54.99	0.075
	placebo	205.10	60.99	190.07	45.835	< 0.001
*P*-value (between)		0.393		0.127		
EFS, %	Centaurea behen	31.38	6.43	34.93	6.09	< 0.001
	placebo	33.67	5.42	34.28	5.43	< 0.001
*P*-value (between)		0.130		0.733		
LV MPI	Centaurea behen	0.71	0.13	0.61	0.12	< 0.001
	placebo	0.62	0.13	0.64	0.11	< 0.001
*P*-value (between)		0.112		0.092		
RV FAC, %	Centaurea behen	31.38	9.26	34.87	9.41	< 0.001
	placebo	28.26	9.81	28.76	8.64	< 0.001
*P*-value (between)		0.778		0.055		
E/e'	Centaurea behen	9.41	4.19	8.81	2.96	< 0.001
	placebo	10.19	4.78	9.30	4.02	0.001
*P*-value (between)		0245		0.318		

Abbreviations: AST, aspartate aminotransferase; ALT, Alanine aminotransferase; ALP, alkaline phosphatase; EFS, ejection fraction calculated by Simpson’s method echocardiography; LV MPI, left ventricular myocardial performance index; RV FAC, right ventricular fractional area change; E/e’, ratio between early diastolic trans mitral flow velocity and early diastolic mitral annular velocity. *P* < 0.05 statistically significant.

**Figure 2:**
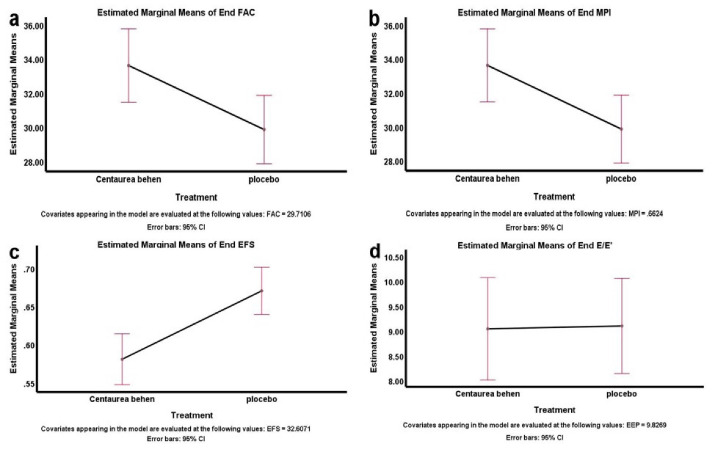
Comparison of the Centaurea behen and placebo groups for the echocardiographic variables. Abbreviations: a, fractional area change (FAC); b, myocardial performance index (MPI); c, ejection fraction calculated by Simpson’s method echocardiography (EFS); d, ratio between early diastolic Trans mitral flow velocity and early diastolic mitral annular velocity (E/e’).

Figure 2 represents simple comparison of Centaurea behen and placebo groups for the studied variables. According to the figure, there was a significant difference between the levels of EFS, MPI, FAC and E/e’ in the 2 groups.

## Discussion

 Heart failure is a progressive disorder that causes damage or loss of strength to the myocardium, resulting in abnormal heart contractions. It is more prevalent with age progression. This clinical trial was designed to investigate effects of Cb on quality of life and heart function of patients with systolic heart failure. In this study, the primary aim was to evaluate quality of life using MLHFQ and 6MWT, and the echocardiographic and laboratory parameters were the other aims.

 There were significant differences in MLHFQ scores within the groups before and after treatment. The result of 6MWT in the Cb group improved significantly after treatment, but not in the placebo group.

 This finding can be attributed to the characteristics of 6MWT as an objective test and a clinical assessment tool of the functional capacity, which is significantly related to the left ventricular systolic function parameters as well as LV MPI.^22^ LV MPI, which measures left ventricular function, was significantly improved in the Cb group after treatment, but not in the placebo group. Other echocardiographic parameters including RV FAC, EFS and E/e’, improved after treatment in both groups, but improvement in Cb group was more significant than placebo group.

 The significance of these parameters, especially 6MWT and LV MPI, can be related to Cb effects. Because of the key role of oxidative stress in the induction of HF,^4^ these improvements and beneficial effects can be related to the reduction of oxidative stress by the phenolic and flavonoid components of Cb like luteoline,^23,24^ circimaritin, jaceosidin, naringenin,^15,25^ Factors such as flavonoids appear to play an important role in inhibiting hydrogen peroxide production by mitochondria,^26^ and,hence, reducing oxidative stress, improving platelet, vascular function,^27^ and scavenging free radicals.^5^ Also phenols scavenge hydrogen peroxide,^5^ and saponins which play a role in the effects of clinical hypocholesterolemia,^28^ and controlling plasma cholesterol,^8^ reduce CVD. Reduction of oxidative stress can lead to strengthening of myocardium contractibility and cardiac performance.^25^ The changes in laboratory parameters were in the normal range which are not clinically significant. ALP, ALT and AST tests can suggest the absence of liver cell damage by Cb.^18^ The use of Cb extract did not increase significantly in some parameters compared to the placebo. This may be due to the non-randomization of those parameters in the results between the two groups or the smallness of the study population. Considering the effect of various factors in heart failure, the limited duration of the treatment period, the lack of long-term follow-up after the intervention and other possible factors, it is not possible to expect a major increase in those parameters.

 An important limitation of this study was the happening of placebo effects during the study, which might have been undetectable due to some possible confounding factors and needs to be carefully, investigated in future studies. Although, the present study was conducted in 2018-19 and classified HF patients into two groups based on the AHA/ACC 2013 guidelines, the current and novel 2022 guidelines classify HF into 4 groups based on EF.

## Conclusion

 Based on the MLHFQ, and 6MWT tests, the consumption of Centaurea behen root extract was associated with significant improvement in the quality of life of patients with systolic heart failure.

## Acknowledgments

 The authors would like to express their gratitude to Prof. Soghrat Faghihzadeh for his valuable statistical and analytical guidance and Ms. Nazari for providing statistical advice. The authors also thank Ms. Emami for her efforts in coordinating the participants. We are also grateful for the participation of all patients and their best cooperation during the research period.

 This article is the result of a research thesis with the Samat code (A-12-594-20) and the ethical approval, approved by Zanjan University of Medical Sciences.

## Competing Interest

 The authors declare that there is no conflict of interest and financial disclosure.

## Ethical Approval

 This clinical trial was performed based on the principles outlined in the Declaration of Helsinki and approved by the ethics committee of Zanjan University of Medical Sciences at number (IR.ZUMS.REC.1396.272). Moreover, the study was registered in the Iranian Registry for Clinical Trials (IRCT20180130038563N1). Written informed consent was obtained from all participants before the study.

## Funding

 This study was funded by Zanjan University of Medical Sciences and also supervised and reviewed the protocol development, data collection, analysis, and reporting process.
